# Mini Review: Nanosheet Technology towards Biomedical Application

**DOI:** 10.3390/nano7090246

**Published:** 2017-08-31

**Authors:** Sheng Zhang, Yuta Sunami, Hiromu Hashimoto

**Affiliations:** 1 Micro/Nano Technology Center, Tokai University, 4-1-1 Kitakaname, Hiratsuka-city, Kanagawa 259-1292, Japan; sunami@tokai-u.jp; 2 Department of Mechanical Engineering, Tokai University, 4-1-1 Kitakaname, Hiratsuka-city, Kanagawa 259-1292, Japan

**Keywords:** nanosheet, fragmented nanosheets, drug delivery, wound treatment

## Abstract

The fabrication technique of ultrathin film (commonly known as nanosheets) has been significantly developed over the years. Due to the mechanical properties of nanosheets, such as high levels of adhesion and flexibility, this made nanosheets the ideal candidate in biomedical applications. In this review, innovative biomedical applications of nanosheets are discussed, which include, drug delivery, wound treatment, and functional nanosheets towards flexible biodevices, etc. Finally, the future outlook of nanosheet technology towards a biomedical application is discussed.

## 1. Introduction

The fabrication technique of ultrathin film (commonly known as nanosheets) is one important subject in nanotechnology, and it has been significantly developed over the years [1]. The unique electronic structure with atomic thickness made two-dimensional (2D) nanosheets to have a tremendously large surface-area-to-thickness ratio, attracted great attentions in a range of areas [2,3,4]. To date, a great amount of two-dimensional nanosheet materials have been developed, especially inorganic nanomaterials as single layer and multilayer nanosheets [5,6]. These unique ultrathin thicknesses and 2D shapes exhibit peculiar physical, chemical, or electronic properties when compared with their bulk state, therefore, demonstrating the potential for various applications, e.g., catalysis, sensing, and energy storage, etc. [7,8,9].

In this mini review, the advances in nanosheet technology towards novel biomedical applications such as drug delivery, wound treatment, and nano-biodevice are discussed. In addition, the future outlook of nanosheet technology is brainstormed.

## 2. Biomedical Application

### 2.1. Drug Delivery

One particular biomedical application for nanosheet is drug delivery, and unsolved critical issues involving how to reach specific targets without harming the normal cells, e.g., anti-cancer drugs. As of today, cancer is one of the biggest challenges facing mankind, and it remains one of the main causes of death worldwide. Nanotechnology is one of the promising routes to attack cancer cells efficiently without undesired severe side effects [10]. In the modern research, nanosheet has been found to provide a potential solution associated with drug delivery, and mainly graphene-based nanosheets (GNS), including graphenes, graphene oxides, and reduced graphene oxides [11,12,13]. One advantage of GNS is the high loading capacities as compared with other nanocarriers for the delivery of chemotherapeutics and biological drugs.

**Figure 1:**
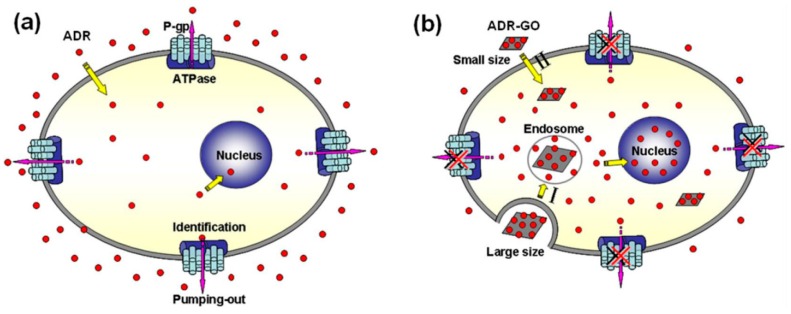
Schematic diagram of (**a**) the adriamycin (ADR) resistance mechanism of surface P-gp in MCF-7/ADR cells and (**b**) the mechanism of Adriamycin graphene oxide (ADR-GO) reversing the ADR resistance [14].

According to the preliminary investigation of Wu et al., graphene oxide (GO) sheet has demonstrated as a carrier for adriamycin (ADR) to reverse drug resistance in breast cancer cells [14]. ADR was loaded onto the GO sheet surface (ADR-GO) with a high drug loading content up to 93.6%. The GO sheet consists of intact graphitic regions interspersed with sp^3^-hybridized carbons on the sheet surface, and sp^2^-hybridized carbons on the aromatic network, as the result, the drug molecules can be loaded effectively (refer to Figure 1). As shown in Figure 1a, ATP hydrolysis releases the energy to P-gp, which then pumps a variety of anticancer drugs, e.g., ADR, out of the cells. Supported by the low endosomal pH trigger, adenosine triphosphate (ADR) can be successfully released into the cytoplasm (refer to Figure 1b). Based on the cell experiments, GO sheet shows significant enhancement of ADR accumulation in MCF-7/ADR cells with much higher cytotoxicity than free ADR. The MCF-7 is one of the most studied cancer cell lines which is named after the Michigan Cancer Foundation (MCF).

**Figure 2:**
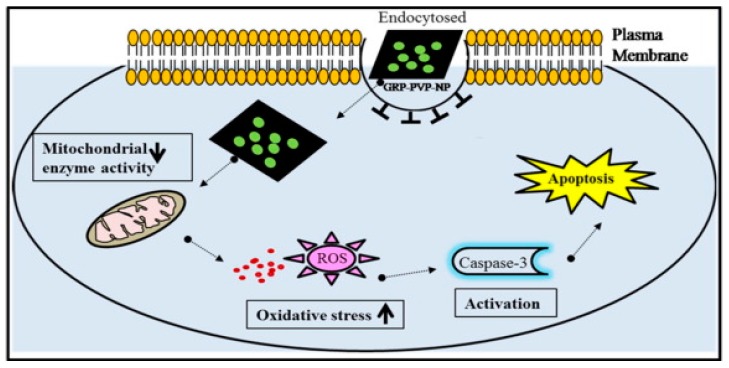
Schematic diagram of potential mechanism with graphene-poly(vinylpyrrolidone) nanoparticle composite (GRP-PVP-NP) induced cytotoxicity in cancer cells [15].

Moreover, a recent study shows that the graphene nanosheet has the dual role as a nanocarrier for the anti-cancer drug, and also acts as an anti-cancer agent [15]. In the research conducted by Tyagi et al. [15], the graphene nanosheets were exfoliated by using poly(vinylpyrrolidone) nanoparticles (PVP-NP), which have higher concentrations of loaded drug caused significant cytotoxicity against both mouse embryonic fibroblasts (NIH-3T3) and human colorectal cancer cells (HCT-116). As shown in Figure 2, the toxic effect of graphene-poly(vinylpyrrolidone) nanoparticle composite (GRP-PVP-NP) against cancer cells is visualized. The GRP-PVP-NP nanosheets served as a nano-carrier for anti-cancer drugs due to the advantage of a high surface area with drugs adhere on both sides of the single sheet. Based on the experimental data, higher concentrations of graphene nanosheets can hamper the growth of cancer cells colony formation ability, and induce cell death through the mechanism of oxidative stress.

### 2.2. Wound Treatment

**Figure 3:**
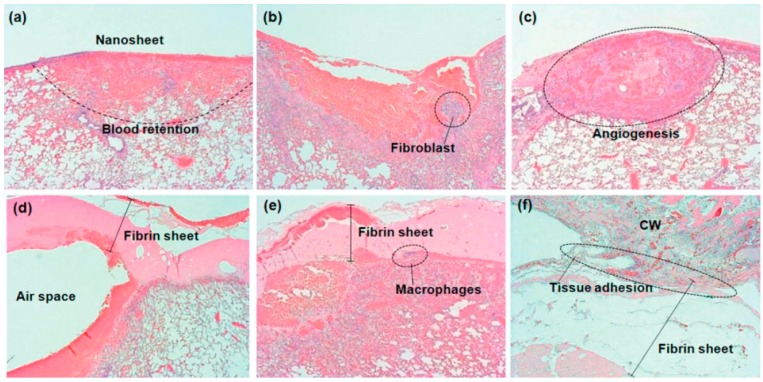
Upper panels correspond to the polysaccharide nanosheet, and lower panels correspond to the fibrin sheet, at 3 h (**a**,**d**), 3 days (**b**,**e**), and 7 days (**c**,**f**) after repair [19].

A series of nanosheet materials possess high levels of adhesiveness and flexibility, and these traits made nanosheets the ideal choice in biomedical applications [16]. For instance, nanosheets composed with versatile clinically used biomaterials can be applied for sealing operations in surgery. Wound healing of skin is a highly coordinated, spatiotemporally regulated process with overlapping phases of hemostasis, inflammation, proliferation, and remodeling [17]. Severe tissue damage or inflammation made suture and ligation technically difficult to conduct in the surgical operations [18]. However, the composition of polyester materials, like poly(l-lactic acid) (PLLA), poly(glycolide), and copolymers enhance nanosheets to have the sufficient adhesive strength to cover the wound area in an relatively easier procedure [19]. The excellent adhesiveness to biological tissue can accelerate the fibrin formation (refer to Figure 3). Moreover, the wound site can be stabilized without eliciting an inflammatory response. According to the research conducted by Okamura et al., a free-standing biocompatible polysaccharide nanosheet composed of chitosan and sodium alginate is fabricated through a spin-coating-assisted layer-by-layer method [20]. A practical working test of the 75 nm polysaccharide nanosheet is demonstrated in vivo on visceral pleural defect model of beagle dogs. This fabricated nanosheet acts as a wound dressing and a method of repairing a gastric incision by sealing instead of surgical suturing. One year later, the research conducted by Fujie et al., demonstrated the nanosheet-type biomaterial can be the potential clinical treatment for repairing a cecal colostomy without chemical bonding agents [21]. The polysaccharide nanosheet was applied to an experimental murine model of cecal puncture, and adhered densely without any adhesive agents. The usage of the polysaccharide nanosheet decreased bacterial extravasation and increased the survival rates of mice.

**Figure 4:**
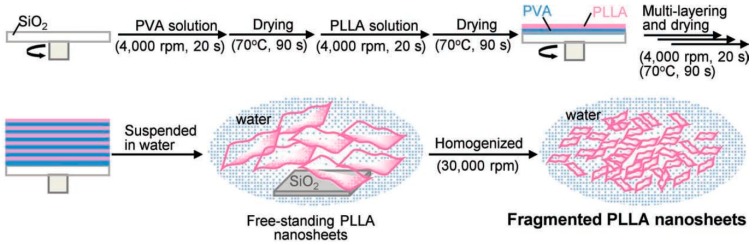
Preparation of fragmented PLLA nanosheets [22].

Moreover, the fragmentation of poly(lactic acid) nanosheets and patchwork treatment for burn wounds is investigated by Okamura et al. [22]. First, 25 freestanding PLLA nanosheets with the thickness of 60 ± 6 nm were homogenized at 30,000 rpm into fragments with the volume of 1.44 × 10^−14^ m^3^, and calculated the specific surface area of fragmented PLLA nanosheets to be 26 m^2^ g^−1^ (refer to Figure 4). Based on the experimental results, the fragmented PLLA nanosheets firmly attached to various interfaces as patchwork with no assistance of any adhesive reagents. The patchwork demonstrates an excellent barrier ability to prevent infection during the treatment of burns for a period of 3 days.

**Figure 5:**
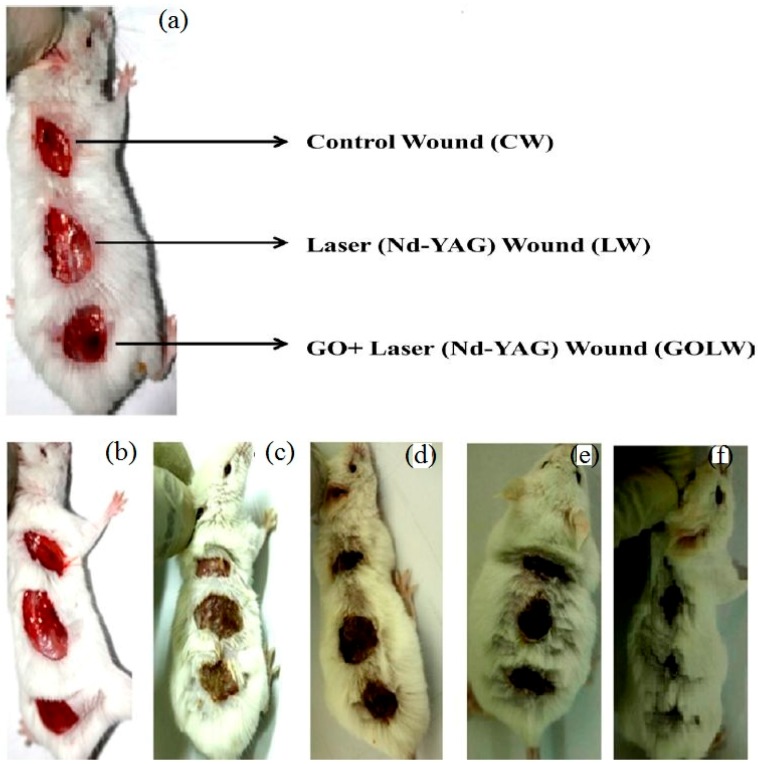
(**a**) Location of wounds, (**b**) fresh wounds on dorsal surface of mice, (**c**) Infection started after 3 days with injection of *S. aureus* on wounds, (**d,e**) condition of wounds after 6 days of treatment, (**f**) wound treatment on the dorsal surface of mice by using GO and Nd-YAG laser [23].

Furthermore, research about graphene oxide nanosheet for an efficient antibacterial, antifungal, and wound treatment is conducted by Khan et al. [23]. The experimental results show the synergistic effect of antibacterial property of GO and neodymium-doped yttrium aluminium garnet (Nd-YAG) laser (1064 nm) for antibacterial and antifungal treatments as a non-invasive method, with a higher efficiency as compared to antibiotics. The new therapy reveals remarkable healing for infection wounds on the dorsal surface of albino mice (adult and male) (refer to Figure 5). A similar investigation is conducted by Ito et al., using silver sulfadiazine-loaded nanosheets to heal a partial-thickness burn injury in a mouse model [24].

### 2.3. Nanosheet towards Biodevices

Nanosheet technology is an innovative and promising approach for health-care practices in surgery, as well as for linking the human body to electronic interfaces and biodevices for future medical applications [25]. Nowadays, electrochemical biosensors can be fabricated with the benefit of nanotechnology offering high sensitivity, real-time detection, low power requirements, and an incredibly small size [26,27].

**Figure 6:**
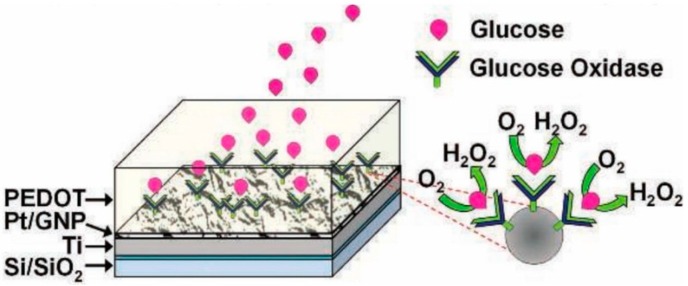
Schematic diagram of GO/PEDOT biofunctionalized PtNP-MGPN glucose biosensor [28].

Researchers have designed a robust multilayered graphene petal nanosheets (MGPN) with platinum nanoparticles for electrochemical biosensing [28]. Pt nanoparticles are electrodeposited onto the MGPNs for the effort of increasing electro-reactivity towards the reduction of oxygen to H_2_O_2_ (refer to Figure 6). The enzyme GO*_x_* is mixed with the conductive polymer poly(3,4-ethylenedioxythiophene (PEDOT) in order to convert the platinum nanoparticles (PtNP)-MGPN electrode into enzymatic biosensors. Outstanding results show that the glucose sensing range is expanded into the physiological concentration levels found in urine, tears, and saliva in addition to blood. Moreover, the electrodeposition of GO*_x_* with PEDOT onto the PtNP modified MGPNs minimizes the robust glucose sensing interference from endogenous electroactive species.

**Figure 7:**
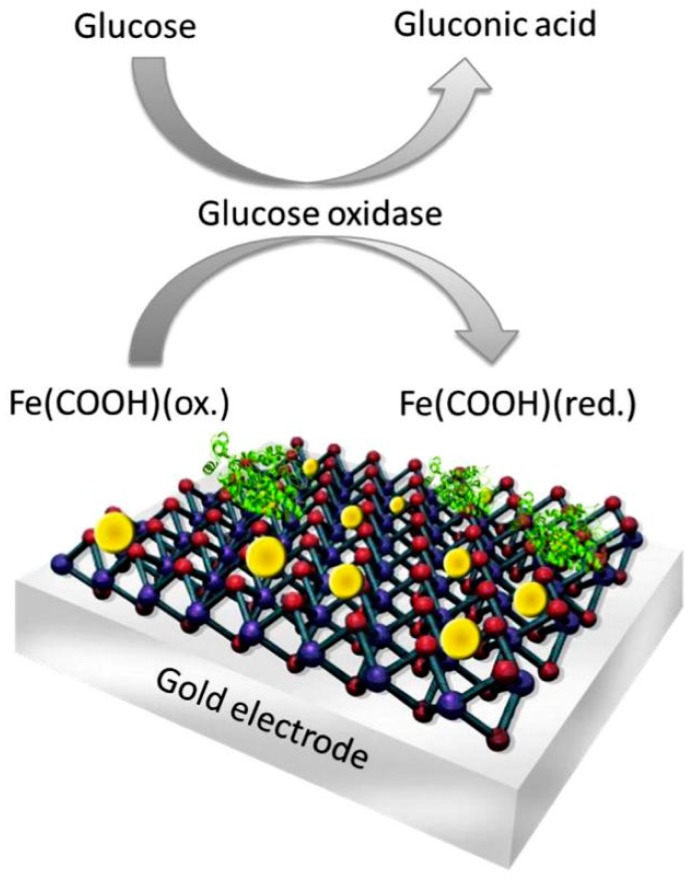
Schematic diagram of Au nanoparticle-structuring on a MoS_2_ nanosheet [29].

In the current state, researchers have combined the electrochemical biosensors with nanosheets into a revolutionary two-dimensional (2D) bioelectronics [29]. The MoS_2_ nanosheets was structured of gold nanoparticles (Au NPs) with an assembly of the enzyme for glucose detection (refer to Figure 7). The fabrication of a two-dimensional biosystem is simple, inexpensive, and high level at the same time. The experimental results have shown that the innovative 2D biosystem has the ability to control bioelectrocatalytic reactions by nanointerface modification.

## 3. Conclusions

Nanosheet technology is an emerging field with a promising future of research. In this mini review, the recent development of nanosheet technology towards biomedical applications is discussed. Sufficient amounts of investigations have proven that nanosheet technology is able to solve the problems and challenges in a series of biomedical areas, e.g., drug delivery, wound treatment, and biodevices. In the future, with a deeper understanding of the cell-material interface, nanosheet involved tissue engineering is expected with even - living organism.
